# Thrombose veineuse mésentérique supérieure compliquant une appendicite méconnue

**DOI:** 10.11604/pamj.2013.14.12.2263

**Published:** 2013-01-08

**Authors:** Rachid El Barni, Abdennasser El Kharras, Mohamed Lahkim, Jawad Fassi Fihri, Abdelhadi Mejdane, Rachid Bouchama, Abdessamad Achour

**Affiliations:** 1 Service de chirurgie générale, Hôpital Militaire Avicenne Marrakech, Maroc; 2 Service d'imagerie médicale. 1er Centre médico-chirurgical Agadir, Maroc

**Keywords:** Thrombose veineuse mésentérique supérieure, appendicite, Superior mesenteric vein thrombosis, appendicitis

## Abstract

La thrombose veineuse mésentérique supérieure (TVMS) peut se présenter selon un mode aigu, subaigu ou chronique. Réputée rare, elle peut être primitive ou secondaire. Les étiologies chirurgicales les plus fréquemment identifiées de TVMS sont la diverticulite colique et l'appendicite aiguë. Les auteurs ont jugé utile de rapporter une observation de TVMS compliquant une appendicite refroidie par les antibiotiques, tout en insistant sur la latence clinique de telle pathologie rendant son diagnostic et son traitement plus difficile.

## Introduction

Les causes chirurgicales les plus fréquemment identifiées de thrombose veineuse mésentérique supérieure (TVMS) sont les diverticulites coliques et l'appendicite aiguë [1]. Le scanner abdominal a prouvé son efficacité en permettant le diagnostic précoce et en identifiant le foyer septique responsable [2]. Une prise en charge immédiate doit être instaurée, elle doit comporter une antibiothérapie, une héparinothérapie et la chirurgie de l'infection intrapéritonéale. Nous rapportons un cas de thrombose veineuse mésentérique supérieure compliquant une appendicite méconnue refroidie par les antibiotiques en ambulatoire.

## Patient et observation

**Figure 1:**
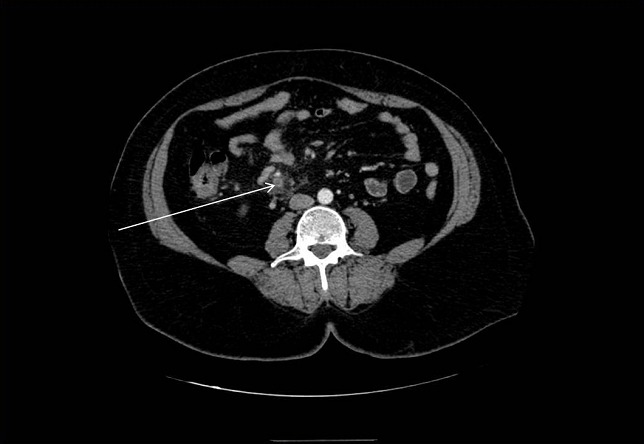
Angio-scanner abdominal montrant une thrombose de la VMS (flèche blanche)

**Figure 2:**
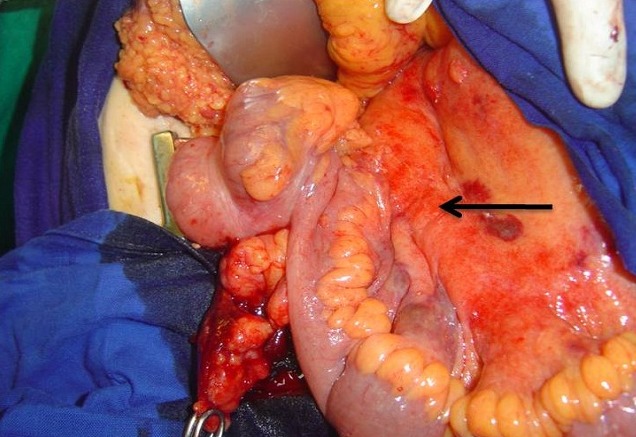
Vue peropératoire montrant un appendice scléreux avec infiltration de la graisse mésentérique adjacente à la VMS (flèche noire)

Mme B. T, âgée de 52 ans, présentait depuis 8 jours des douleurs abdominales hypogastriques avec vomissements et traitait par antibiothérapie en ambulatoire. L'examen clinique trouvait une patiente fébrile à 38,5° avec une sensibilité abdominale généralisée et une défense de la fosse iliaque droite. Le bilan biologique trouvait un syndrome inflammatoire non spécifique. Le bilan hépatique montrait une légère cytolyse. L’échographie abdominale permettait d’éliminer une cholécystite et suspecter une appendicite. L'angio-scanner abdominal montrait l'aspect d'une appendicite aiguë avec une importante réaction inflammatoire de la graisse mésentérique compliquée d'une thrombose étendue de la veine mésentérique supérieure (VMS). Cette thrombose respectait la portion terminale de la VMS, le tronc spléno-mésaraïque et le tronc porte (Figure 1). Une héparinothérapie associée à une antibiothérapie étaient démarrées. Une laparotomie médiane était réalisée en urgence, et l'exploration chirurgicale trouvait un appendice scléreux avec mésentère infiltré et épaississement des dernières anses grêliques sans nécrose intestinale (Figure 2). Des prélèvements bactériologiques, une appendicectomie avec drainage étaient jugées suffisantes. L'examen bactériologique des prélèvements effectués en peropératoire montrait une Escherichia Coli. L’évolution clinique était satisfaisante. Un bilan de thrombophilie revenait sans anomalies. Le relais par les anti-vitamines K (AVK) était débuté dès la 48ème heure et l'héparinothérapie était administrée pendant 8 jours. Une anticoagulation par AVK était poursuivie pendant six mois au bout desquels un angio-scanner abdominal avait montré la reperméabilisation de la VMS.

## Discussion

Une thrombose veineuse mésentérique peut être aiguë, subaiguë ou chronique [3–5]. Rhee et al. ont défini par forme aiguë toute thrombose veineuse mésentérique diagnostiquée moins de quatre semaines après le début des signes [6]. Réputée rare, l'incidence réelle de TVM reste inconnue [7]. Il s'agit d'une pathologie grave avec une mortalité supérieure à 50%. Dans la majorité des cas, la pyléphlébite est la conséquence de l'extension de proche en proche de thrombophlébites suppurées [8]. Les étiologies les plus courantes des pyléphlébites sont des affections aiguës ascendantes d'un organe abdominal comme la nécrose pancréatique, l'appendicite, la cholécystite aiguë, la diverticulite aiguë ou perforée [8]. Cliniquement, certains symptômes sont plus spécifiques quand ils précèdent le syndrome septique à l'origine de la TVM. La fièvre, les frissons, l'hyperleucocytose et la douleur abdominale avec défense sont les signes initiaux [1, 9]. Le foyer septique initial est souvent silencieux [1, 9]. Biologiquement, les signes de sepsis sont marqués. La radiographie de l'abdomen sans préparation n'est pas contributive [4]. L’échographie abdominale est peu performante [10]. Actuellement, la tomodensitométrie abdominale avec injection de produit de contraste constitue le moyen le plus efficace pour reconnaître une thrombose veineuse mésentérique supérieure. Sa sensibilité est de 90 à 100% [3, 11]. Elle visualise soit directement le thrombus dans la veine mésentérique sous forme d'une image lacunaire endoluminale due au défaut de remplissage lié au thrombus entouré par un liséré périphérique hyperdense [12], soit des signes indirects moins spécifiques comme un épaississement de la paroi intestinale et/ou un épanchement intrapéritonéal. Elle permet aussi de préciser les limites de la thrombose, de montrer une éventuelle extension aux veines collatérales, de rechercher une présence d'air dans le système porte ou la veine mésentérique ainsi que d’écarter une autre maladie à l'origine de la symptomatologie digestive [10]. L’échographie doppler aurait une bonne sensibilité pour la détection du thrombus ou d'une anomalie de flux dans la veine mésentérique mais il requiert un échographiste expérimenté et la rentabilité est moindre que celle de la tomodensitométrie [3, 13]. Il constitue un bon examen pour la surveillance de la reperméabilisation du système porte. Quant à l'imagerie par résonance magnétique, sa sensibilité apparaît superposable à celle de la tomodensitométrie [14] mais son coût nécessite de la réserver aux contre-indications à l'injection de produits iodés. Le traitement précoce associant antibiothérapie à large spectre et anticoagulants à dose efficace est impératif [8]. Si l'objectif de l'anticoagulation systémique est de stabiliser le thrombus afin d’éviter sa progression dans le réseau veineux mésentérique, l'adjonction d'un agent thrombolytique par voie générale ou régionale peut s'avérer être un complément thérapeutique très utile notamment dans les TVM aiguës étendues ou complètes avec un risque élevé d’évolution vers la nécrose intestinale étendue et le décès du patient [15]. Le relais par AVK se fait dès que la certitude d'absence d’évolution vers la nécrose ischémique est acquise. Sa durée est habituellement de six mois à un an, sauf si un trouble de la coagulation a été identifié comme étant la cause de l'ischémie [5]. La chirurgie traite le foyer infectieux responsable sans délai. L’évolution se fait généralement vers la disparition de la thrombose. Elle peut se faire cependant vers un cavernome portal justifiant de surveiller ces patients [9].

## Conclusion

Il faut suspecter le diagnostic d'appendicite en cas de thrombose de la VMS. Si le diagnostic d'appendicite ne peut pas être fait sur des arguments cliniques, la pratique systématique d'un scanner à la recherche de signes d'appendicite, de même que la relecture ou la répétition de cet examen en cas de doute est nécessaire.
